# Hydrogen ion concentration and coronary artery bypass graft surgery with and without cardiopulmonary bypass

**DOI:** 10.1186/1749-8090-8-184

**Published:** 2013-08-20

**Authors:** Cher Shiong Chuah, Rachael Kirkbride, R Peter Alston, Joanne Irons

**Affiliations:** 1 College of Medicine and Veterinary Medicine, University of Edinburgh, 47 Little France Crescent, Edinburgh EH16 4TJ, UK; 2 Department of Anaesthesia, Critical Care and Pain Medicine, Royal Infirmary of Edinburgh, 51 Little France Crescent, Edinburgh EH16 4SA, UK; 3 Department of Anaesthesia, Papworth Hospital, Papworth Everard, Cambridge CB23 3RE, UK

**Keywords:** Cardiopulmonary bypass, Hydrogen ion concentration, Strong ion difference, Intravascular fluids, Coronary artery bypass grafting surgery

## Abstract

**Background:**

Acidosis during cardiopulmonary bypass (CPB) has been related to the strong ion difference (SID) and the composition of intravascular fluids that are administered. Less intravascular fluids tend to be administered during off- than on-pump CABG and should influence the degree of acidosis that develops. This study aimed to explore the role of CPB in the development of acidosis by comparing changes in hydrogen ion concentration ([H^+^]) and electrolytes in patients undergoing on- and off-pump coronary artery bypass graft (CABG) surgery.

**Methods:**

Eighty two patients had arterial blood gas measurements pre-operatively, following CABG and at approximately 0600 h the morning after surgery. Carbon dioxide tension (PaCO_2_) and concentrations of sodium, potassium, chloride, [H^+^], bicarbonate and haemoglobin were measured and strong ion difference calculated. Data was analysed using mixed repeated-measures analysis of variance.

**Results:**

Intra-operatively, mean SID decreased more in the on- compared to the off-pump group (4.0 mmol/L, 95% confidence interval 2.8-5.3 mmol/L, p < 0.001). Neither [H^+^] or PaCO_2_ changed significantly and there were no significant difference between the groups. By the morning following surgery, [H^+^] and PaCO_2_ had both increased (p < 0.001) and difference in SID had disappeared (p = 0.17).

**Conclusion:**

Despite significant differences in changes in SID, there were no differences in [H^+^] between patients during or after CABG surgery whether performed on- or off-pump. This may be have been the result of greater haemodilution in the on- compared to the off-pump group, compensating for change in SID by reducing the concentration of weak acids. Although it was associated with significantly greater decrease in SID, CPB was not associated with any significant increased risk of acidosis.

## Background

Although originally performed without it, coronary artery bypass graft (CABG) surgery has traditionally been undertaken using cardiopulmonary bypass (CPB) since the 1970s. Since the 1990s there has been a resurgence of interest in performing CABG surgery off-pump that is without the use of CPB [1]. The motivation for undertaking CABG surgery off-pump was that avoiding CPB might reduce haemodynamic instability and the incidences of adverse events such as stroke and myocardial injury [2,3]. Another adverse event associated with CPB is the development of metabolic acidosis [4]. However, the aetiology of the metabolic acidosis remains unclear. Recent work has suggested that Stewart’s quantitative acid–base approach might allows a better understanding of the causes of this acidosis [5,6].

Conventionally, lactate accumulation secondary to hypoperfusion was thought to be the prime driver of the metabolic acidosis. However, using Stewart’s hypothesis, acidosis during CPB has been related to a decrease in strong ion difference (SID) during CPB [7]. The normal SID is 42 mmol/L [8]. Indeed, Hayhoe and colleagues demonstrated that the lactate concentration actually decreased during CPB [9]. Research indicates suggested that the solutions used to prime the CPB circuit influences metabolic acidosis [7,9,10]. Indeed, Himpe and colleagues found that metabolic acidosis could be limited by using priming solutions that have a high SID while Alston and colleagues demonstrated that changing the priming solution from Ringer’s to Hartmann’s which increases the SID of the prime from −2 to 27 mmol/L, was associated with less metabolic acidosis [4,11]. In addition, the administration of intravascular fluids that alter SID also affect [H^+^] [12-14].

Off-pump CABG surgery avoids the intravascular infusion of large volumes of fluids used to prime the CPB circuit and so should reduce acidosis compared to on-pump CABG surgery. Indeed, it has been suggested that off-pump CABG surgery is associated with a lower incidence of metabolic acidosis [15]. Therefore, the aim of this study is to compare changes in electrolyte concentration and their influence of [H+] that occur in patients undergoing CABG surgery on- and off-pump.

## Methods

Ethical approval by the South East Scotland Research Ethics Committee (11/WS/0110) and management approval (2011/R/CAR/20) were granted for the study to be conducted at the Royal Infirmary of Edinburgh. Patient recruitment took place from January to May 2012. All patients scheduled to undergo isolated CABG surgery during this period were approached by researchers. Written consent was obtained from patients who wished to participate. Patients undergoing combined procedures and emergency surgery were exclusion criteria.

The study design was observational and patient allocation to on-or off-pump CABG surgery was at the discretion of the surgeon. Similarly, anaesthetic technique and the choice and volume of intravascular fluids that were administered were at the discretion of the anaesthesiologist. For patients on CPB, the priming solution for the circuit was 1,800 ml of Hartmann’s, 200 ml of mannitol 20%, 50 ml of sodium bicarbonate 8.4% and 10 ml of heparin (10,000 iu). During CPB, mean arterial pressure (MAP) was maintained between 60–80 mmHg. Hypotension was corrected with boluses of metaraminol 0.5 mg if required.

As part of anaesthetic and post-operative care, routine arterial blood samples were obtained by operating department practitioners in theatre and by nurses in cardiac intensive treatment unit. Measurements were retrieved retrospectively from three routinely calibrated, RapidPoint 500 blood gas analysers (Siemens, Germany). Three blood gas measurements were made on each patient. A pre-operative measurement was taken after insertion of an arterial line before induction of anaesthesia. A measurement was performed immediately after CABG and the administration of protamine for heparin reversal. A third measurement was taken the morning after surgery at approximately 0600 hours. The blood samples were analysed for concentrations of sodium, potassium, chloride, hydrogen ion, bicarbonate, haemoglobin and partial pressure of carbon dioxide. SID and base excess (BE) were calculated using the formula in Appendices I and II [16]. Patient and surgical characteristics as well as all intravascular fluids administered during surgery were recorded.

Statistical analysis was carried out using SPSS v20 (Armonk, New York, USA). Differences in patient characteristics were explored using student’s *t*-test. Differences in surgical characteristics were compared using Mann–Whitney *U*-test. Correlations between variables were explored using Pearson’s correlation coefficient. Analysis of variance (ANOVA) was the main statistical test applied for the measurements to compare the difference between the on- and off-pump group as well as difference over the three time points. The probability of post-hoc tests were corrected for multiple testing using the Bonferroni correction and a p-value of less than 5% was considered significant.

A population sample size calculation was made to determine the number of patients required for each arm of the study. Previous research in this hospital showed that patients undergoing on-pump CABG surgery had a mean [H+] of 36 nmol/L with a standard deviation of 6 nmol/L [4]. Based on this mean and standard deviation, it was initially calculated that 36 patients were needed in each group to detect an effect size of 4 nmol/L (α = 0.05, power = 0.80) which was deemed to be a clinical important change in [H+]. Due to the limited number of procedures performed, only 26 patients who underwent off-pump CABG surgery were recruited into the study. However, this shortfall in patients did not compromise the statistical power of the study. A retrospective power calculation based on the actual values obtained found that this study was able to detect a change of 4 nmol/L with a power of 0.84 (α = 0.05).

## Results

Eighty-two patients were approached: one patient declined to participate. Fifty five patients underwent on-pump CABG surgery and 26 patients in the off-pump CABG surgery. Patient characteristics are similar and there were no significant differences between the groups (Table 1). Operative characteristics differed between the groups. Off-pump patients had shorter durations of surgery and stay in the critical care areas and less intravascular fluids administered (Table 2).

**Table 1:** Characteristics of patients undergoing on- and off-pump CABG surgery

	**On-pump CABG surgery (n = 55)**		**Off-pump CABG surgery (n = 26)**		
	**Mean**	**95% Confidence interval**	**Mean**	**95% Confidence interval**	
**Age**	67.0	64.8-69.1	63.4	59.8-66.9	p = 0.10
**Height (m)**	1.72	1.70-1.74	1.71	1.67-1.75	p = 0.51
**Weight (kg)**	83.5	79.2-87.8	87.3	81.3-93.3	p = 0.33
**BMI**	28.1	26.9-29.3	29.8	28.0-31.6	p = 0.21
**Gender**	**n**	**Percentage**	**n**	**Percentage**	
**Male**	45	82%	21	81%	
**Female**	10	18%	5	19%	

Where BMI is body mass index.

**Table 2:** Operative and recovery characteristics

	**On-pump CABG surgery (n = 55)**		**Off-pump CABG surgery (n = 26)**		
	**Mean**	**95% Confidence interval**	**Mean**	**95% Confidence interval**	
**Duration of surgery (min)**	215	204-227	186*	174-198	p < 0.05
**Duration of CPB (min)**	103	94-111	-	-	
**Total intravascular fluid volume (ml)**	3570	3330-3811	2190*	1816-2563	p < 0.001
	**Median**	**Interquartile range**	**Median**	**Interquartile range**	
**Duration of tracheal intubation (h)**	15	8-20	8*	6-12	p < 0.05
**Duration of critical care stay (h)**	47	23-86	24*	20-48	p < 0.05

Where *CPB* cardiopulmonary bypass and *:significant difference from on-pump group, p < 0.05 (Mann–Whitney *U*-test).

### Hydrogen ion concentration, carbon dioxide tension and strong ion difference

Mean concentrations of sodium, potassium, chloride, strong ion difference, hydrogen ion, bicarbonate, carbon dioxide tension, haemoglobin concentration are presented in Table 3.

**Table 3:** Mean blood concentrations in on- and off-pump CABG surgery groups pre-operatively, following CABG and the morning following surgery

		**On-pump (n = 55)**			**Off-pump (n = 26)**		
		**Mean**	**95% Confidence interval**		**Mean**	**95% Confidence interval**	
			**Lower**	**Upper**		**Lower**	**Upper**
**Na**^**+ **^**(mmol/L)**	Pre-operatively	139.5	138.9	140.2	139.4	138.7	140.1
	Post-CABG	135.8^‡^	135.1	136.5	137.6^‡^	136.7	138.5
	Next-morning	137.2^‡^	136.4	138.0	135.3^‡^	134.5	136.1
**K**^**+ **^**(mmol/L)**	Pre-operatively	4.03	3.95	4.10	4.18	4.01	4.34
	Post-CABG	4.81^‡^	4.66	4.97	4.25^‡^	4.06	4.44
	Next-morning	4.70	4.59	4.80	4.66	4.48	4.84
**Cl**^**- **^**(mmol/L)**	Pre-operatively	105.3	104.7	105.9	105.2	104.0	106.3
	Post-CABG	111.0^‡^	110.2	111.7	108.2^‡^	106.9	109.5
	Next-morning	106.8^‡^	105.9	107.7	104.0^‡^	102.7	105.3
**SID (mEq/L)**	Pre-operatively	38.2	37.5	38.9	38.4	37.3	39.6
	Post-CABG	29.7^‡^	29.0	30.3	33.7^‡^	32.4	35.0
	Next-morning	35.1	34.5	35.7	36.0	34.7	37.2
**[H**^**+**^**] (nmol/L)**	Pre-operatively	38.1	37.4	38.8	39.0	37.1	40.9
	Post-CABG	39.8	38.3	41.3	40.1	37.7	42.5
	Next-morning	41.9	40.7	43.2	43.8	42.0	45.7
**HCO**_**3**_^**- **^**(mmol/L)**	Pre-operatively	24.9	24.2	25.5	24.6	23.7	25.5
	Post-CABG	22.6	22.0	23.2	23.2	22.1	24.2
	Next-morning	23.9	23.2	24.7	23.2	22.4	24.0
**PaCO**_**2 **_**(kPA)**	Pre-operatively	5.23	5.07	5.39	5.30	5.05	5.56
	Post-CABG	4.93	4.73	5.12	5.15	4.81	5.49
	Next-morning	5.60	5.39	5.81	5.82	5.48	6.16
**Hb (g/L)**	Pre-operatively	13.8	13.5	14.1	14.4	13.6	15.1
	Post-CABG	10.1^‡^	9.7	10.5	12.1^‡^	11.2	12.9
	Next-morning	10.5^‡^	10.1	10.8	12.0^‡^	11.4	12.6
**BE (mEq/L)**	Pre-operatively	0.38	−0.20	1.00	0.15	−0.90	1.21
	Post-CABG	−1.96	−2.66	−1.26	−1.25	−2.41	−0.09
	Next-morning	−0.75	−1.38	−0.13	−1.17	−2.13	−0.22

Where *Na*^*+*^ sodium, *K*^*+*^ potassium, *Cl*^*-*^ chloride, *SID* strong ion difference, *[H*^*+*^*]* hydrogen ion concentration, *HCO*_*3*_^*-*^ bicarbonate, *PaCO*_*2*_ arterial carbon dioxide tension, *Hb* haemoglobin concentration, *CABG* coronary artery bypass grafting, next-morning: morning following surgery, ^‡^ p < 0.05.

**Figure 1:**
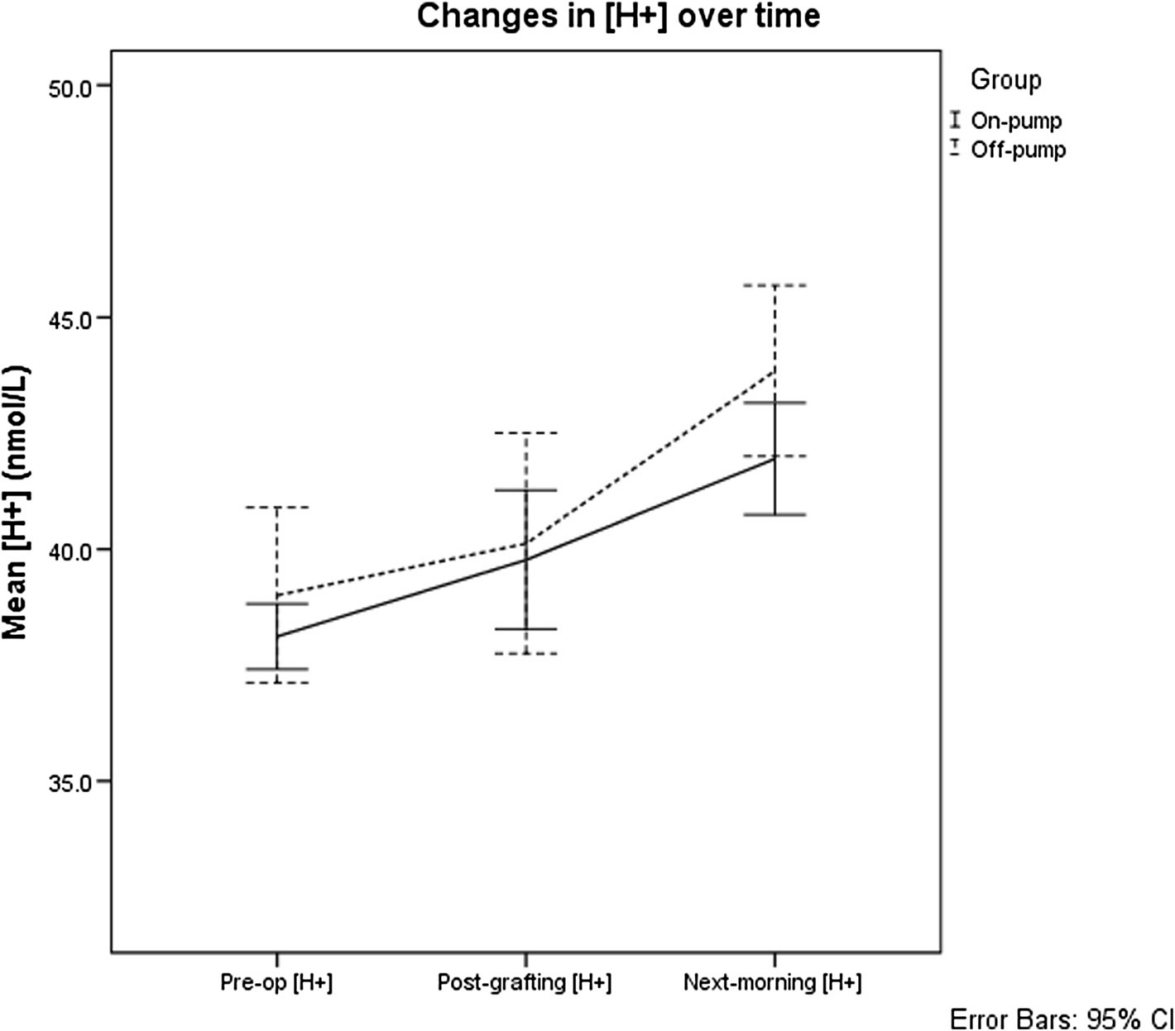
**Changes in hydrogen ion concentration in patients undergoing on- and off-pump coronary artery bypass grafting surgery.** Mean hydrogen ion concentration [H+] and 95% confidence intervals for patients undergoing on- and off-pump patients over time. Where pre-op: pre-operatively, post-grafting: following coronary artery bypass grafting and next-morning: approximately 0600 on the morning following surgery.

**Figure 2:**
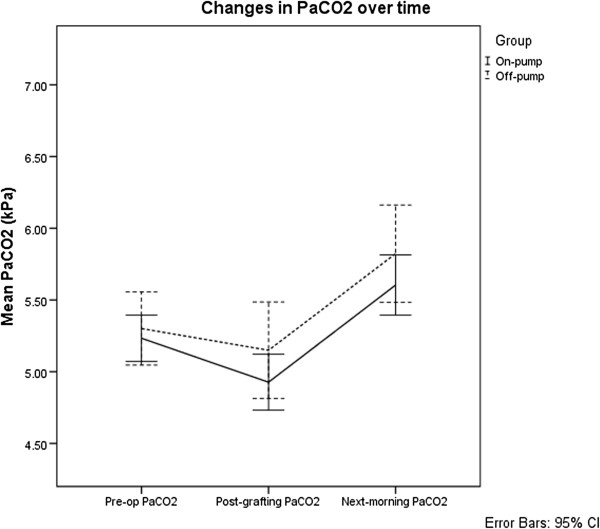
**Changes in arterial carbon dioxide tension in patients undergoing on- and off-pump coronary artery bypass grafting surgery.** Mean arterial carbon dioxide tensions (PaCO_2_) and 95% confidence intervals for patients undergoing on- and off-pump patients over time. Where pre-op: pre-operatively, post-grafting: following coronary artery bypass grafting and next-morning: approximately 0600 on the morning following surgery.

**Figure 3:**
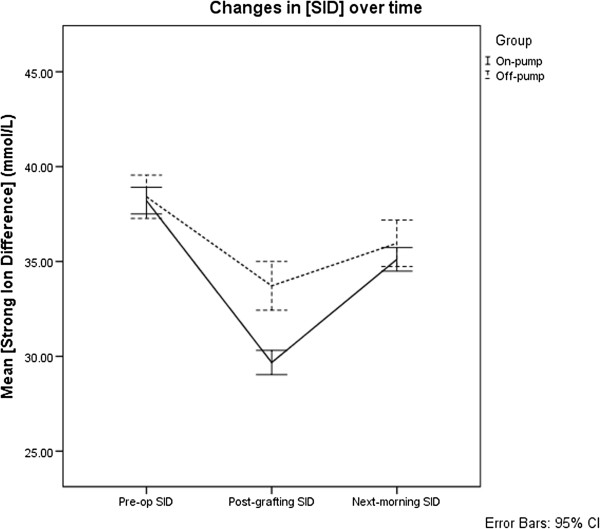
**Changes in strong ion difference in patients undergoing on- and off-pump coronary artery bypass grafting surgery.** Mean concentrations of strong ion difference ([SID]) and 95% confidence intervals for patients undergoing on- and off-pump patients over time. Where pre-op: pre-operatively, post-grafting: following coronary artery bypass grafting and next-morning: approximately 0600 on the morning following surgery.

Mixed repeated-measures ANOVA and post-hoc analysis applying the Bonferroni correction were used to analyse [H^+^], PaCO_2_ and SID. The results for these are shown and summarised in Figures 1, 2 and 3. There were no detectable differences in [H^+^] (F (1,79) = 1.79, p = 0.185) and PaCO_2_ (F (1,79) = 1.91, p = 0.171) between the groups. Both [H^+^] and PaCO_2_ showed no significant change over the course of the operation. However, on the morning following surgery, [H^+^] was significantly higher than both pre-operative (mean difference = 4.3 nmol/L, p < 0.001) and post-CABG [H^+^] (mean difference = 3.0 nmol/L, p < 0.001). PaCO_2_ on the morning following surgery was also significantly higher than both pre-operative (mean difference = 0.45 kPa, p < 0.001) and post-CABG (mean difference = 0.68 kPa, p < 0.001) time points.

There were significant differences in SID over time (F(2,158) = 185.9, p < 0.001) as well as between groups (F(1,79) = 12.46, p < 0.001). Post-hoc analysis showed that mean SID differed significantly over all time points (Table 4). Over the course of surgery, both groups experienced a decrease in SID with on-pump patients experiencing a greater decrease than off-pump patients, resulting in a significant difference at the post-CABG measurement (mean difference = 4.0 mmol/L, 95% CI 2.8-5.3 mmol/L, p < 0.001). By the morning following surgery, the differences between on- and off-pump groups became non-significant and SID returned towards pre-operative values (Figure 3).

**Table 4:** Changes in strong ion difference over time

**Time points**	**Mean difference**	**95% CI**
Pre-operatively to post-CABG	6.62*	5.72-7.51
Post-CABG to morning following surgery	3.84*	3.09-4.60
Pre-operatively to morning following surgery	2.77*	1.90-3.65

Where CABG is coronary artery bypass grafting, * p < 0.001.

### Intravascular fluids and tracheal extubation

**Figure 4:**
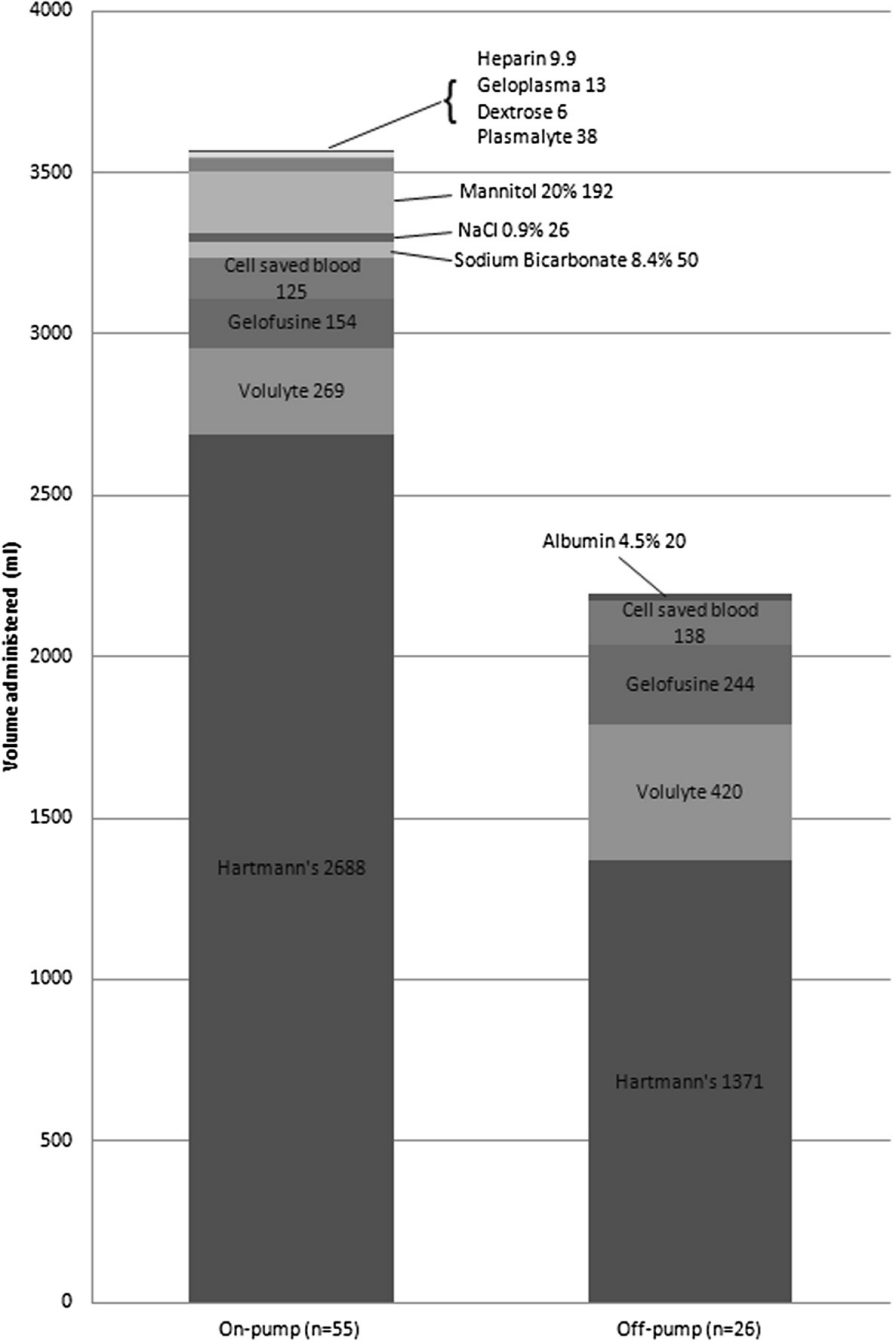
Average composition of fluids given to patients undergoing on-pump and off-pump coronary artery bypass graft surgery.

The compositions of fluids administered to both groups were similar (Figure 4). The mean total volumes of fluid administered intravascularly during surgery was 3,570 ml (SD 739 ml) and 2,190 ml (SD 877 ml) in the patients undergoing on- and off-pump groups respectively with a mean difference of 1,380 ml (95% CI 970-1780 ml).

Patients whose tracheas were extubated before the measurement on the morning following surgery had a significantly higher mean PaCO_2_. Students *t*-test found that mean PaCO_2_ was 5.90 kPa in patients who had undergone tracheal extubation and significantly (p < 0.01) higher compared 5.40 kPa in patients whose tracheas were still intubated. Tracheal extubation also correlated with an increase in PaCO_2_ (Pearson r = 0.31, p < 0.01).

### Predictors of hydrogen ion concentration

Univariate correlations were performed comparing changes in [H^+^] and SID to changes in PaCO_2_, Hb and SID. [H^+^] was found to be significantly correlated with PaCO_2_ (r = 0.53, p < 0.01) and SID (r = −0.35, p < 0.01). SID was significantly correlated with Hb (r = 0.48, p < 0.01). A multivariate linear regression model was then applied to assess the strength and independence of the relationships between the factors that correlated significantly with [H^+^]. A significant model was found using PaCO_2_ and SID as predictors of [H^+^] (adjusted r square = 0.48, F = 33.0, p < 0.001). The standardised coefficient β for PaCO_2_ was 0.624 and SID was −0.473 (both p < 0.001). Haemoglobin, which was used as a marker of haemodilution, was not entered into the regression model as no significant univariate correlation was found between SID and Hb.

## Discussion

### Intra-operative hydrogen ion concentration, carbon dioxide tension and strong ion difference

Over the course of surgery, there was a significant decrease in SID, with patients in the on-pump group demonstrating a greater decrease compared with the off-pump group. This was found to be related to the total volume of fluid administered intravascularly. According to Stewart’s hypothesis, we would expect such a significant change in SID to directly affect [H^+^] or for a change in PaCO_2_ to neutralise the change in SID. However, [H^+^] and PaCO_2_ did not change over the course of surgery.

Therefore, an explanation for the lack of effect of SID on [H^+^] may be that the change in SID was compensated by changes in total weak acid concentration (A_TOT_). A_TOT_ is mainly composed of albumin as the principal extracellular weak acid [17]. During CPB, patients develop a dilutional hypoalbuminaemia as a result of intravascular fluid administration [10,11,18]. This hypoalbuminaemia induces a metabolic alkalosis, which counteracts the acidosis induced by the administration of intravascular fluids [7,10].

To support this explanation, this study used haemoglobin concentration as a marker of haemodilution as any dilution of albumin and other blood proteins can be indirectly inferred from changes in haemoglobin [7]. Indeed, SID was found to correlate with haemoglobin which suggests that a decrease in SID is accompanied with a corresponding haemodilution. Hence, a possible explanation for why the difference in SID between the groups did not affect [H^+^] is the more fluid that is administered, the more SID decreases and at the same time, A_TOT_ is diluted. Therefore the acidifying effects of a decrease in SID may be balanced by the alkalizing effects of a dilution in A_TOT_. In hindsight, measuring lactate might have improved the interpretation of the results.

### Post-operative changes in hydrogen ion concentration

Post-operatively, [H^+^] and PaCO_2_ significantly increased in both groups and there were no significant differences between them. In addition, changes in SID tended to resolve towards the baseline measurement and the difference in SID between on- and off-pump groups disappeared. Together these findings suggest that the majority of the change in [H^+^] post-operatively can be attributed to changes in PaCO_2_.

### On- compared to off-pump CABG surgery

This study demonstrates that patients undergoing off-pump surgery received less intravascular fluids and therefore, experience less severe disturbances of sodium, potassium, chloride, bicarbonate and so SID in the immediate post-CABG period compared to on-pump patients. However, these differences tend to narrow or disappear by the morning following surgery which is between 12–18 hours postoperatively.

### Implications

The findings of this study confirm that changes in SID are related to intravascular fluids that are administered. The findings cannot preclude the possibility that an acidosis develops by the end of surgery. However, following completion of CABG and protamine administration, there is no significant change in [H^+^] from baseline or between on- and off-pump groups, despite significant differences in SID. Ultimately, the changes in SID resolved relatively quickly, such that by the morning following SID is almost back to its baseline measurement. Therefore, two things can be inferred: firstly, while fluids affect SID, this may not necessarily lead on to acidosis and secondly, the use of cardiopulmonary bypass seems to have little impact on the development of acidosis.

## Conclusion

This study found that there were no significant differences in [H^+^] between patients undergoing on- or off-pump CABG surgery. The use of CPB is associated with more intravascular fluids and leads to a greater decrease in SID. However, this does not impact acidosis. SID normalised post-operatively and the difference between groups at the end of CPB diminished by the morning following surgery.

## Appendix

### Appendix I

Formula for calculating strong ion difference:

$$\mathrm{SID}=\left[Na^{+}\right]+\left[K^{+}\right]-\left[Cl^{-}\right]$$

### Appendix II

Formula for calculating base excess:

$$\begin{array}{l} B.E.=0.02786\times \mathrm{PaC}O_{2}\left(\mathrm{mmHg}\right)\times 10^{\left(\mathrm{pH}-6.1\right)}\\ \;+13.77\times \mathrm{pH}-124.58 \end{array}$$

## Abbreviations

CPB: Cardiopulmonary bypass; CABG: Coronary artery bypass grafting; SID: Strong ion difference; BE: Base excess; ANOVA: Analysis of variance; [H+]: Hydrogen ion concentration; PaCO2: Arterial carbon dioxide tension; ATOT: Total weak acid concentration; Hb: Haemoglobin; MAP: Mean arterial pressure.

## Competing interests

The authors declare that they have no competing interests.

## Authors’ contributions

CSC carried out patient recruitment, data collection, performed the statistical analyses and drafted the manuscript. RK carried out patient recruitment, data collection, reviewed the statistics and the draft manuscript. RPA conceived of the study, supervised its implementation, provided statistical guidance and reviewed the manuscript. JI assisted in patient recruitment, provided advice and reviewed the statistics and the manuscript. All authors read and approved the final manuscript.

## References

[B1] Jansen EW Borst C Lahpor JR Gründeman PF Eefting FD Nierich A Coronary artery bypass grafting without cardiopulmonary bypass using the octopus method: results in the first one hundred patients J Thorac Cardiovasc Surg 1998 116 1 60 67 10.1016/S0022-5223(98)70243-0 9671898

[B2] Shroyer AL Grover FL Hattler B Collins JF McDonald GO Kozora E On-pump versus off-pump coronary-artery bypass surgery N Engl J Med 2009 361 19 1827 1837 10.1056/NEJMoa0902905 19890125

[B3] Puskas JD Williams WH Duke PG Staples JR Glas KE Marshall JJ Off-pump coronary artery bypass grafting provides complete revascularization with reduced myocardial injury, transfusion requirements, and length of stay: a prospective randomized comparison of two hundred unselected patients undergoing off-pump versus conventional coronary artery bypass grafting J Thorac Cardiovasc Surg 2003 125 4 797 808 10.1067/mtc.2003.324 12698142

[B4] Alston RP Theodosiou C Sanger K Changing the priming solution from Ringer’s to Hartmann’s solution is associated with less metabolic acidosis during cardiopulmonary bypass Perfusion 2007 22 6 385 389 10.1177/0267659108089142 18666740

[B5] Prough DS White R Acidosis associated with perioperative saline administration: dilution or delusion? Anesthesiol 2000 93 5 1167 1169 10.1097/00000542-200011000-00005 11046200

[B6] Stewart PA Modern quantitative acid–base chemistry Can J Physiol Pharmacol 1983 61 12 1444 1461 10.1139/y83-207 6423247

[B7] Alston RP Cormack L Collinson C Metabolic acidosis developing during cardiopulmonary bypass is related to a decrease in strong ion difference Perfusion 2004 19 3 145 152 10.1191/0267659104pf751oa 15298421

[B8] Gattinoni L Carlesso E Cadringher P Caironi P Strong ion difference in urine: new perspectives in acid–base assessment Crit Care 2006 10 2 137 10.1186/cc4890 16677408 PMC1550906

[B9] Hayhoe M Bellomo R Liu G McNicol L Buxton B The aetiology and pathogenesis of cardiopulmonary bypass-associated metabolic acidosis using polygeline pump prime Intensive Care Med 1999 25 7 680 685 10.1007/s001340050930 10470571

[B10] Liskaser FJ Bellomo R Hayhoe M Story D Poustie S Smith B Role of pump prime in the etiology and pathogenesis of cardiopulmonary bypass–associated acidosis Anesthesiol 2000 93 5 1170 1173 10.1097/00000542-200011000-00006 11046201

[B11] Himpe D Neels H De Hert S Van Cauwelaert P Adding lactate to the prime solution during hypothermic cardiopulmonary bypass: a quantitative acid–base analysis Br J Anaesth 2003 90 4 440 445 10.1093/bja/aeg084 12644414

[B12] Scheingraber S Rehm M Sehmisch C Finsterer U Rapid saline infusion produces hyperchloremic acidosis in patients undergoing gynecologic surgery Anesthesiol 1999 90 5 1265 1270 10.1097/00000542-199905000-00007 10319771

[B13] Waters JH Miller LR Clack S Kim JV Cause of metabolic acidosis in prolonged surgery Crit Care Med 1999 27 10 2142 2146 10.1097/00003246-199910000-00011 10548196

[B14] Rahn H Body temperature and acid–base regulation (review article) Lung 1974 151 2 87 94 10.1007/BF02097155 4615310

[B15] Lancey RA Soller BR Vander Salm TJ Off-pump versus on-pump coronary artery bypass surgery: a case-matched comparison of clinical outcomes and costs Heart Surg Forum 2000 3 4 277 281 11178287

[B16] Calculated bicarbonate & base excess http://www-users.med.cornell.edu/~spon/picu/calc/basecalc.htm

[B17] Figge J Mydosh T Fencl V Serum proteins and acid–base equilibria: a follow-up J Lab Clin Med 1992 120 5 713 719 1431499

[B18] Hatherill M Salie S Waggie Z Lawrenson J Hewitson J Reynolds L Hyperchloraemic metabolic acidosis following open cardiac surgery Arch Dis Child 2005 90 12 1288 1292 10.1136/adc.2005.078006 16159902 PMC1720224

